# Tetracycline-modifying enzyme *Sm*TetX from *Stenotrophomonas maltophilia*

**DOI:** 10.1107/S2053230X23005381

**Published:** 2023-07-05

**Authors:** Martin Malý, Petr Kolenko, Jan Stránský, Leona Švecová, Jarmila Dušková, Tomáš Koval’, Tereza Skálová, Mária Trundová, Kristýna Adámková, Jiří Černý, Paulína Božíková, Jan Dohnálek

**Affiliations:** a Institute of Biotechnology, Czech Academy of Sciences, v.v.i., BIOCEV, Průmyslová 595, 252 50 Vestec, Czech Republic; b Faculty of Nuclear Sciences and Physical Engineering, Czech Technical University in Prague, Břehová 7, 115 19 Prague 1, Czech Republic; Centro Nacional de Biotecnología – CSIC, Spain

**Keywords:** FAD-dependent monooxygenases, tetracycline, antibiotic resistance

## Abstract

The resistance of the emerging human pathogen *Stenotrophomonas maltophilia* to tetracycline antibiotics mainly depends on multidrug efflux pumps and ribosomal protection enzymes. However, the genomes of several strains of this Gram-negative bacterium code for a FAD-dependent monooxygenase (*Sm*TetX) homologous to tetracycline destructases. This protein was recombinantly produced and its structure and function were investigated. Activity assays using *Sm*TetX showed its ability to modify oxytetracycline with a catalytic rate comparable to those of other destructases. *Sm*TetX shares its fold with the tetracycline destructase TetX from *Bacteroides thetaiotaomicron*; however, its active site possesses an aromatic region that is unique in this enzyme family. A docking study confirmed tetracycline and its analogues to be the preferred binders amongst various classes of antibiotics.

## Abstract 2

*Stenotrophomonas maltophilia* codes for a tetracycline-modifying FAD-dependent monooxygenase that shares an overall fold with the tetracycline destructase TetX and possesses a unique active site within this enzyme family.

## 1. Introduction

Antibiotic resistance has become a global health problem of continually increasing magnitude (Lewis, 2020 ▸; Murray *et al.*, 2022 ▸). *Stenotrophomonas maltophilia* is an opportunistic multidrug-resistant pathogen (Brooke, 2012 ▸) which is responsible for an increasing number of infections (Brooke, 2014 ▸; Chang *et al.*, 2015 ▸). This Gram-negative bacterium attacks immunocompromised hosts in hospital environments, particularly patients affected by cystic fibrosis, lung cancer or chronic obstructive pulmonary disease (Esposito *et al.*, 2017 ▸). Apart from respiratory tract infections, *S. maltophilia* can also be associated with bacteremia, urinary tract infections, meningitis, endocarditis, osteomyelitis and biliary sepsis. However, the efficacy of antibiotic therapy is often limited due to the low susceptibility of this pathogen to a broad spectrum of drugs, including tetracycline antibiotics (Sánchez, 2015 ▸; Gajdács & Urbán, 2019 ▸).

The molecular structure of tetracycline antibiotics comprises a linear fusion of four rings with various functional groups attached (Chopra & Roberts, 2001 ▸). These molecules are used in the treatment of a broad spectrum of bacterial infections by binding to the 30S subunit of the ribosome, which inhibits protein synthesis (Jenner *et al.*, 2013 ▸; Brodersen *et al.*, 2000 ▸). Tetracycline antibiotics have been widely used against both Gram-positive and Gram-negative bacteria in medicine, and also in the food industry to protect plants, aquacultures and animal growth.

**Figure 1:**
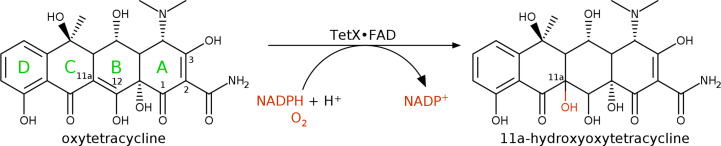
Simplified reaction scheme of the degradation of oxytetracycline catalyzed by the tetracycline destructase TetX. The four individual rings of the molecule are labelled in green. The C atoms susceptible to modification by tetracycline destructases are labelled with their numbers.

The excessive use of antibiotics has contributed to the development of various mechanisms of drug resistance in bacteria. In the case of tetracycline antibiotics, the leading resistance mechanisms are the overexpression of chromosomally encoded multidrug efflux pumps (Zhao *et al.*, 2018 ▸), ribosomal protection proteins and enzymatic modification of antibiotic molecules (Nguyen *et al.*, 2014 ▸; Grossman, 2016 ▸; He *et al.*, 2015 ▸). Multidrug efflux pumps are essential for the strong resistance of *S. maltophilia* to tetracycline antibiotics. There is also some evidence that *S. maltophilia* may have a tetracycline-specific efflux pump (He *et al.*, 2015 ▸). The first two mechanisms, efflux pumps and ribosomal protection, have been suppressed by the design of new semi-synthetic variants of tetracycline antibiotics (for example tigecycline; Yaghoubi *et al.*, 2022 ▸) using a targeted selection of functional groups bound to the tetracycline ring (Sum *et al.*, 1994 ▸). However, the third mechanism, enzymatic modification, which was discovered relatively recently (Yang *et al.*, 2004 ▸; Moore *et al.*, 2005 ▸), still represents a serious threat. The enzymes of the tetracycline destructase family (EC 1.14.13.231; Cheng *et al.*, 2022 ▸; Fang *et al.*, 2020 ▸; Markley & Wencewicz, 2018 ▸) are capable of covalent modification of the tetracycline scaffold, which causes degradation to inactive and unstable compounds, for example 11a-hydroxy-oxytetracycline (Yang *et al.*, 2004 ▸): see the reaction scheme in Fig. 1 ▸. The structural and functional properties of such enzymes from other bacteria have been elucidated by determining crystal structures, for example of TetX from *Bacteroides thetaiotaomicron* (PDB entries 2y6r and 4a6n; Volkers *et al.*, 2011 ▸, 2013 ▸), TetX2 from *B. thetaiotaomicron* (PDB entry 3p9u; Walkiewicz *et al.*, 2011 ▸), TetX4 from *Escherichia coli* (PDB entry 7epw; Cheng *et al.*, 2021 ▸), TetX6 from *Chryseobacterium oncorhynchi* (PDB entries 8er0 and 8er1; Kumar *et al.*, 2023 ▸), TetX7 from *Pseudomonas aeruginosa* (PDB entry 6wg9; Gasparrini *et al.*, 2020 ▸), Tet(56) from *Legionella longbeachae* (PDB entry 5tum; Park *et al.*, 2017 ▸) and Tet(50), Tet(51) and Tet(55) from an uncultured bacterium (PDB entries 5tue, 5tuk and 5tul; Park *et al.*, 2017 ▸). The tetracycline destructases are classified as class A FAD-dependent monooxygenases (FDOs), which cover numerous proteins with diverse purposes and functions that primarily catalyze the hydroxylation of aromatic substrates. Their characteristic structural feature is the presence of a FAD-binding domain that tightly binds the FAD prosthetic group. Their catalytic function is dependent on NADH or NADPH co-factors.

To the best of our knowledge, the degradation of tetracycline antibiotics by *S. maltophilia* has not yet been studied. Antibiotic-modifying enzymes for β-lactams and aminoglycosides have been reported in *S. maltophilia*, but not for tetracyclines (Gil-Gil *et al.*, 2020 ▸). However, several strains of *S. maltophilia* code for a FAD-dependent monooxygenase (NCBI Reference Sequence WP_049406473; O’Leary *et al.*, 2016 ▸) that shares 28% sequence identity with the tetracycline destructase TetX. We carried out a biophysical and structural analysis of the recombinant form of this enzyme to elucidate whether it could contribute to the resistance of *S. maltophilia* to tetracycline antibiotics. The nucleotide sequence coding for the enzyme has been found in clinical isolates from India, Australia, Europe (Esposito *et al.*, 2017 ▸; Lira *et al.*, 2017 ▸) and the USA (Pak *et al.*, 2015 ▸; Roach *et al.*, 2015 ▸). Our results show its close structural and functional relationship to the tetracycline destructase enzyme family.

## 2. Materials and methods

### 2.1. Bioinformatic analysis and target selection

A search for nucleotide sequences coding for proteins similar to tetracycline destructases was performed on the NCBI database (NCBI Resource Coordinators, 2016 ▸) using *Protein BLAST* (Boratyn *et al.*, 2013 ▸). The amino-acid sequence of TetX from *B. thetaiotaomicron* (Volkers *et al.*, 2011 ▸; PDB entry 2y6r) was used as a search template. The sequence of *Sm*TetX, a putative FAD-dependent monooxygenase from *S. maltophilia* strain AB550 (Permala *et al.*, 2018 ▸) with NCBI Reference Sequence WP_049406473 (O’Leary *et al.*, 2016 ▸; Arita *et al.*, 2021 ▸) included in GenBank entry CP028899 (Glady-Croue *et al.*, 2018 ▸), shares 28% sequence identity and 41% sequence similarity with TetX according to *EMBOSS Needle* (Rice *et al.*, 2000 ▸).

### 2.2. Recombinant expression

The target amino-acid sequence (NCBI Reference Sequence WP_049406473) was back-translated and codon usage was optimized for expression in *E. coli* with *OPTIMIZER* (Puigbò *et al.*, 2007 ▸). A plasmid encoding the *Sm*TetX protein including a 6×His tag at the N-terminus of the translated protein was synthesized and cloned into the pET-28a(+)-TEV expression vector via NdeI and BamHI restriction sites by GenScript, USA. The cleavage site for Tobacco etch virus (TEV) protease was placed between the His tag and the target *Sm*TetX sequence (Supplementary Fig. S1).

The plasmid was transformed into competent *E. coli* strain Lemo21 (DE3) cells (New England Biolabs) using the heat-shock method. Cell precultures in LB medium were incubated in a shaker at 32°C and 180 rev min^−1^ overnight. The medium was supplemented with 50 µg ml^−1^ kanamycin and 25 µg ml^−1^ chloramphenicol. 10 ml preculture was added to 1 l Power Broth medium (Molecular Dimensions, catalogue No. MD12-106) and the cell culture was incubated at 30°C and 150 rev min^−1^ until the OD_600_ reached ∼0.5. Expression was induced with 1 m*M* isopropyl β-d-1-thiogalactopyranoside (IPTG) at 20°C for 16 h. The cells were harvested after centrifugation of the culture at 4°C and 5000*g* for 30 min.

### 2.3. Purification and characterization

The cell pellet was homogenized in lysis buffer consisting of 50 m*M* Tris–HCl, 500 m*M* NaCl, 30 m*M* imidazole pH 8, protease-inhibitor cocktail (Sigma–Aldrich, catalogue No, P8849) and DNase I (Sigma–Aldrich, catalogue No. D4263). The cells were gently lysed by sonication (Qsonica Q700). After centrifugation at 40 000*g* for 30 min and filtration using a Puradisc 25 mm PTFE 1.0 µm (Whatman), the clarified lysate was loaded onto an equilibrated 5 ml HisTrap FF column (GE Healthcare) for Ni–NTA affinity chromatography. The column was then washed with 50 m*M* Tris–HCl, 500 m*M* NaCl, 30 m*M* imidazole pH 8 and the protein was eluted using the same buffer with a step gradient to 170 m*M* imidazole using an ÄKTApurifier FPLC system (GE Healthcare/Amersham Biosciences) at 10°C.

The buffer was exchanged to 50 m*M* Tris–HCl, 150 m*M* NaCl pH 8, 0.5 m*M* dithiothreitol (DTT), 1 m*M* ethylenediaminetetraacetic acid using a 3 kDa cutoff Nanosep centrifugal device (Pall Corporation); the final protein concentration was 1 mg ml^−1^. TEV cleavage was performed at 4°C for 16 h using TEV protease at a concentration of 0.05 mg ml^−1^; TEV protease production and the enzymatic reaction were carried out according to a standard protocol (Tropea *et al.*, 2009 ▸). Tag-free *Sm*TetX was separated on a 5 ml HisTrap FF column (GE Healthcare).

Size-exclusion chromatography was performed using a Superdex 75 Increase 10/300 GL column (GE Healthcare) equilibrated with buffer consisting of 25 m*M* Tris–HCl, 150 m*M* NaCl pH 8. Sample purity was checked by sodium dodecyl sulfate–polyacrylamide gel electrophoresis (Supplementary Fig. S2); the gel was made using the TGX FastCast Acrylamide Kit 12% (Bio-Rad) and a Colour Prestained Protein Standard, Broad Range marker (New England Biolabs) and was stained with InstantBlue (Expedeon). The protein sample was characterized by nanoscale differential scanning fluorimetry (NanoDSF) carried out in a Prometheus NT.48 (NanoTemper) and isoelectric focusing (IEF; Novex pH 3–10 IEF, 5% polyacrylamide gel, ThermoFisher Scientific; analysis was performed using the protocol recommended by the manufacturer). The UV–Vis spectrum of the protein (Supplementary Fig. S3) at a concentration of 3 mg ml^−1^ was measured in 100 m*M* [tris(hydroxymethyl)methylamino]­propanesulfonic acid (TAPS) pH 8.5 in a quartz cuvette (optical path 10 mm) using a Specord 50 Plus spectrophoto­meter (Analytik Jena).

### 2.4. Structural mass spectrometry

For this analysis, *Sm*TetX was alkylated by iodoacetamide at a final concentration of 30 m*M*. After 30 min incubation in the dark, trypsin was added to a concentration of 0.1 µg ml^−1^ and the reaction mixture was incubated overnight at 37°C.

Samples were analysed using a liquid-chromatography system (Agilent 1200 series, Agilent Technologies) connected to a timsToF Pro PASEF mass spectrometer equipped with a CaptiveSpray ion source (Bruker Daltonics) operated in positive data-dependent mode. 5 µl peptide mixture was injected using an autosampler onto a C18 trap column (UHPLC Fully Porous Polar C18 2.1 mm ID, Phenomenex). After 5 min of trapping at a flow rate of 20 µl min^−1^, peptides were separated on a C18 column (Luna Omega 3 µm Polar C18 100 Å, 150 × 0.3 mm, Phenomenex) using a linear 35 min water–acetonitrile gradient from 5 to 35%(*v*/*v*) acetonitrile at a flow rate of 4 µl min^−1^. Both the trap and analytical columns were heated to 50°C. Parameters from the PASEF method for standard proteomics were used for the timsTOF Pro settings.

The raw data were processed using the *PeaksStudio *10.0 software (Bioinformatics Solutions, Canada). The search parameters were set up as follows: enzyme − trypsin (specific), carbamidomethylation and oxidation of methionine as a variable modification. Data were searched against the *Sm*TetX sequence (Supplementary Fig. S4).

### 2.5. Small-angle X-ray scattering

After size-exclusion chromatography (described above), the protein in the most concentrated fraction (4.4 mg ml^−1^) was transferred to buffer composed of 25 m*M* bis-Tris, 150 m*M* NaCl pH 6.5 via overnight dialysis using Slide-A-Lyzer MINI Dialysis Devices, 3.5K MWCO (Thermo Scientific). Protein samples were centrifuged at 15 000 rev min^−1^ for 15 min prior to small-angle X-ray scattering (SAXS) measurements. Two samples were analysed: protein with reducing agent (30 m*M* DTT) and a control sample of protein without any additive. Data were collected at the Centre of Molecular Structure (BIOCEV), Czech Republic using a MetalJet C2+ X-ray source with a gallium anode (Excillum), SAXSpoint 2.0 (Anton Paar) and an EIGER R 1M detector (Dectris). Scattering images were processed in *Aares*, custom-made software for the in-house source developed by J. Stránský. Further analysis and processing was performed using the *ATSAS* software package (Manalastas-Cantos *et al.*, 2021 ▸). Parameters and statistics relating to the measurements are listed in Supplementary Table S1. The scattering images are available at https://doi.org/10.5281/zenodo.7348780. The processed data including *ab initio* shape determination have been deposited in the SASBDB database under accession codes SASDPW7 and SASDPV7 for sample with and without reducing agent, respectively (Supplementary Fig. S5).

### 2.6. Activity assay

A spectrophotometric assay was performed in 96-well plates (BRAND, Wertheim, Germany) at 25°C in triplicate using a similar setup as reported previously (Forsberg *et al.*, 2015 ▸; Gasparrini *et al.*, 2020 ▸; Moore *et al.*, 2005 ▸; Rudra *et al.*, 2018 ▸; Yang *et al.*, 2004 ▸). Enzymatic reactions of the substrates (0.5 m*M* oxytetracycline, Sigma–Aldrich, catalogue No O5875; 0.5 m*M* NADPH, Sigma–Aldrich, catalogue No. 10621692001) were carried out with 0.1 µ*M* *Sm*TetX in 100 m*M* TAPS buffer pH 8.5 in a total volume of 100 µl. Changes in the optical density were monitored using a Spark microplate reader (Tecan) in the UV–Vis spectrum with 1 min intervals.

### 2.7. Crystallization, diffraction data collection and processing

The optimal protein buffer for crystallization was selected according to screening using the nanoscale differential scanning fluorimetry method conducted on a Prometheus NT.48 (NanoTemper). The protein sample was transferred to buffer consisting of 20 m*M* bis-Tris, 50 m*M* NaCl pH 6.5 and concentrated to 10 mg ml^−1^ using a 3 kDa cutoff Nanosep centrifugal device (Pall Corporation). When searching for the optimal crystallization condition, we used several commercial crystallization screens, including our acidic screen (Fejfarová *et al.*, 2016 ▸). 96-well crystallization plates were set up by a Gryphon crystallization robot (Art Robbins) using the sitting-drop vapour-diffusion method and were stored and monitored in an RI1000 protein crystallization imager (Formulatrix) at a temperature of 20°C; the protein:reservoir ratios were 2:1, 1:1 and 1:2 in a 0.3 µl drop. The initial crystallization hits from the MORPHEUS screen (Molecular Dimensions; Gorrec, 2009 ▸) were further optimized in hanging drops using the microseeding method and Additive Screen (Hampton Research). The final condition consisted of 12%(*w*/*v*) PEG 8000, 24%(*v*/*v*) ethylene glycol, 60 m*M* sodium nitrate, 60 m*M* disodium hydrogen phosphate, 60 m*M* ammonium sulfate, 100 m*M* MES–imidazole pH 6.5, 4% acetone; the protein:reservoir ratio was 2:1 in a 1.5 µl drop.

The crystals were harvested in LithoLoops (Molecular Dimensions) and vitrified in liquid nitrogen without cryoprotection owing to the presence of ethylene glycol at a sufficient concentration in the crystallization conditions. Diffraction data were collected on beamline 14.1 of the BESSY II synchrotron-radiation source (Helmholtz Zentrum Berlin, Germany; Mueller *et al.*, 2015 ▸) using a mini-kappa goniometer and a PILATUS 6M detector (Dectris) under the control of *MXCuBE* (Oscarsson *et al.*, 2019 ▸). The data set was processed in *XDSGUI* (Kabsch, 2010 ▸) and initially scaled in *AIMLESS* from the *CCP*4 suite (Evans & Murshudov, 2013 ▸; Agirre *et al.*, 2023 ▸). A limited range of diffraction images (2400 images, corresponding to 240° of the total rotational angle) were processed due to an increase in *R*_meas_ per image in the final stage of data collection. The diffraction data exhibited high anisotropy: the suggested diffraction limit according to the criterion of *I*/σ(*I*) being higher than 1.5 varied from 2.69 to 1.96 Å for different directions in reciprocal space, as reported in *AIMLESS*. After anisotropy correction with *STARANISO* (Tickle *et al.*, 2018 ▸), the phase problem was solved with a combination of *MoRDa* (Vagin & Lebedev, 2015 ▸; Krissinel *et al.*, 2018 ▸) and *Phaser* (McCoy *et al.*, 2007 ▸) at 2.4 Å resolution. The crystal structure of AbsH3 (Clinger *et al.*, 2021 ▸; PDB entry 6n04) was used as a template; its FAD-binding domain and substrate-binding domain were placed individually into the unit cell. The structure model was refined with *REFMAC*5 (Kovalevskiy *et al.*, 2018 ▸) using restraints for FAD from *AceDRG* (Long *et al.*, 2017 ▸) and manually edited as in Švecová *et al.* (2021 ▸); harmonic restraints were applied to several water molecules to avoid clashes. Manual modifications and real-space refinement were carried out in *Coot* (Emsley *et al.*, 2010 ▸). The high-resolution diffraction limit (1.95 Å) was determined by the paired refinement protocol with *PAIREF* (Karplus & Diederichs, 2012 ▸; Malý *et al.*, 2020 ▸, 2021 ▸). Regions in the model that were difficult to interpret due to a lack of signal were resolved using a combination of polder maps (Liebschner *et al.*, 2017 ▸), composite omit maps (Terwilliger *et al.*, 2008 ▸) and feature-enhanced maps (Afonine *et al.*, 2015 ▸) from the *Phenix* package (Liebschner *et al.*, 2019 ▸). The final structure was refined using all reflections and was validated with *Coot*, *MolProbity* (Williams *et al.*, 2018 ▸) and the wwPDB validation service (Berman *et al.*, 2003 ▸). Data-collection, processing and refinement statistics are shown in Table 1 ▸. The diffraction images are available from the Structural Biology Data Grid (https://data.sbgrid.org/) at https://doi.org/10.15785/SBGRID/956. The coordinates and structure-factor amplitudes were deposited in the PDB with accession code 8aq8. The presented structure alignments and calculations of root-mean-square deviation (r.m.s.d.) were carried out in *PyMOL* 2.5 (Schrödinger). The crystal structure and its similarity to other protein structures were investigated with *ProFunc* (Laskowski *et al.*, 2005 ▸), *PISA* (Krissinel & Henrick, 2007 ▸), *STRIDE* (Heinig & Frishman, 2004 ▸), *PDBsum* (Laskowski *et al.*, 2018 ▸), *PDBeFold* (Krissinel & Henrick, 2004 ▸), *VAST* (Madej *et al.*, 2014 ▸) and *DALI* (Holm, 2020 ▸).

We also attempted to solve the structure of *Sm*TetX in complex with a tetracycline antibiotic using soaking and co-crystallization, without success.

### 2.8. Molecular docking

The initial geometries of the studied antibiotics were obtained from ChEMBL (Mendez *et al.*, 2019 ▸) and PubChem (Kim *et al.*, 2021 ▸) in SDF format. When necessary, the structures were converted to 3D coordinates and H atoms were added with *Open Babel* version 3.1.1 (O’Boyle *et al.*, 2011 ▸). The geometry of the ligands was further optimized with *ORCA* version 5.0.3 (Neese *et al.*, 2020 ▸) at the dispersion-corrected DFT (RI-TPSS-D3/SVP) level of theory in two steps. Firstly, hydrogen positions were optimized while heavy-atom coordinates were kept fixed, followed by full all-atom geometry optimization. For the ligand–protein docking, the structures were converted to PDBQT format (partial atomic charges and atomic types assigned) using the *prepare_receptor*4 and *prepare_ligand*4 Python scripts from *MGLTools* 1.5.7 (Sanner, 1999 ▸) for the protein and ligands, respectively. Docking was performed with *AutoDock Vina* version 1.2.3 (Eberhardt *et al.*, 2021 ▸; Trott & Olson, 2010 ▸) using a 40 × 40 × 40 Å box centred around the C2′ atom of FAD with focus on the *re* site. For each ligand, a set of predicted most stable complex geometries was obtained from the results of 20 independent *AutoDock Vina* runs. The poses were visualized in *PyMOL* 2.5 (Schrödinger). The flexible side-chain docking into the *re* site included protein residues Thr47, Leu48, Asp49, His51, Lys102, Glu104, Phe181, Ser201, Phe203, Phe212, Gln214, Tyr224, Val301 and Trp354 found in the vicinity of the antibiotic ligands.

## 3. Results

### 3.1. Recombinant form of *Sm*TetX

The FAD-dependent monooxygenase from *S. maltophilia* (NCBI Reference Sequence WP_049406473) was recombinantly expressed in *E. coli* cells (Supplementary Fig. S2). The protein consists of 364 amino-acid residues and a FAD prosthetic group that causes the characteristic yellow colour of the sample. The UV–Vis spectrum of *Sm*TetX shows a dominant oxidized state of FAD, but the reduced state is also partially present (Supplementary Fig. S3). The estimated melting temperature of *Sm*TetX, as determined using NanoDSF, is ∼48°C and its isoelectric point is 5.4, as determined using IEF. The structure of the *Sm*TetX monomer based on the crystal structure (described below) fits both SAXS data sets well with a χ^2^, as calculated by *CRYSOL*, of close to 1 (Supplementary Table S1 and Supplementary Fig. S5). Thus, *Sm*TetX is a monomer in solution. Mass-spectrometric analysis of *Sm*TetX peptide fragments shows a basically complete coverage of the construct sequence and the absence of disulfide bridges, as all of the cysteine residues in the protein were observed to be alkylated (Supplementary Fig. S4).

### 3.2. Activity against oxytetracycline

**Figure 2:**
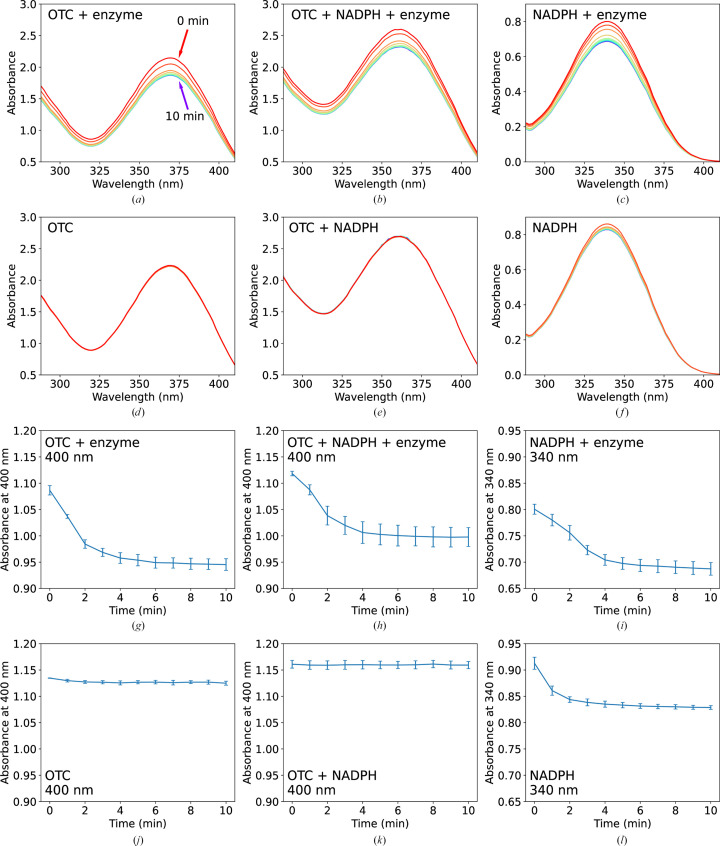
*Sm*TetX causes changes in the UV–Vis spectrum of oxytetracycline (OTC) and NADPH. The initial concentrations of the reagents, which are listed in each panel, were [OTC] = 0.5 m*M*, [NADPH] = 0.5 m*M* and [enzyme] = 0.1 µ*M*. The individual enzymatic assays were performed in parallel in 100 m*M* TAPS pH 8.5. (*a*–*f*) Absorbance scans are plotted at 1 min intervals over a time course of 10 min, represented by a rainbow colour gradient [from red through yellow to blue as shown in (*a*)]. Background absorbance of the buffer was subtracted. (*g*–*l*) Monitoring of the decrease in absorbance of OTC (400 nm) and NADPH (340 nm); standard errors of the mean from measurements in triplicate are shown.

*Sm*TetX was tested for activity against oxytetracycline (OTC). The analysed substrates, OTC and nicotinamide adenine dinucleotide phosphate (NADPH), exhibit absorption peaks at 368 and 340 nm, respectively. Thus, the activity against OTC was monitored similarly to previous studies (Forsberg *et al.*, 2015 ▸; Gasparrini *et al.*, 2020 ▸; Moore *et al.*, 2005 ▸; Rudra *et al.*, 2018 ▸; Yang *et al.*, 2004 ▸) using the decrease in absorption at 400 nm. *Sm*TetX causes a decrease in the absorbance at 400 nm in the absorption spectrum of OTC that indicates enzymatic activity (Figs. 2 ▸ *b* and 2 ▸ *h*). The changes in the OTC+enzyme spectra appear in the presence, and even in the absence, of the NADPH co-factor (Figs. 2 ▸ *a* and 2 ▸ *g*). The estimated turnover numbers for OTC modification by the enzyme, based on the absorbance change at 400 nm during the first 2 min, for OTC and for OTC with NADPH are *k*_cat_ ≃ 2.6 ± 0.7 and *k*_cat_ ≃ 2.0 ± 0.7 s^−1^, respectively. The estimated turnover numbers for NADPH oxidation by the enzyme, based on the absorbance change at 340 nm during the first 2 min, for NADPH and for NADPH with OTC are *k*_cat_ ≃ 1.6 ± 0.8 and *k*_cat_ ≃ 2.1 ± 0.7 s^−1^, respectively. Both reactions appear to be saturated after several minutes as no further decrease in absorbance can be observed. The protein is stable under the reaction conditions used, as was confirmed by NanoDSF; therefore, the loss of activity cannot be attributed to enzyme degradation. The enzyme also oxidizes NADPH in the absence of a tetracycline antibiotic (Figs. 2 ▸ *c* and 2 ▸ *i*). This reaction also achieves saturation in several minutes.

### 3.3. Crystal structure

**Figure 3:**
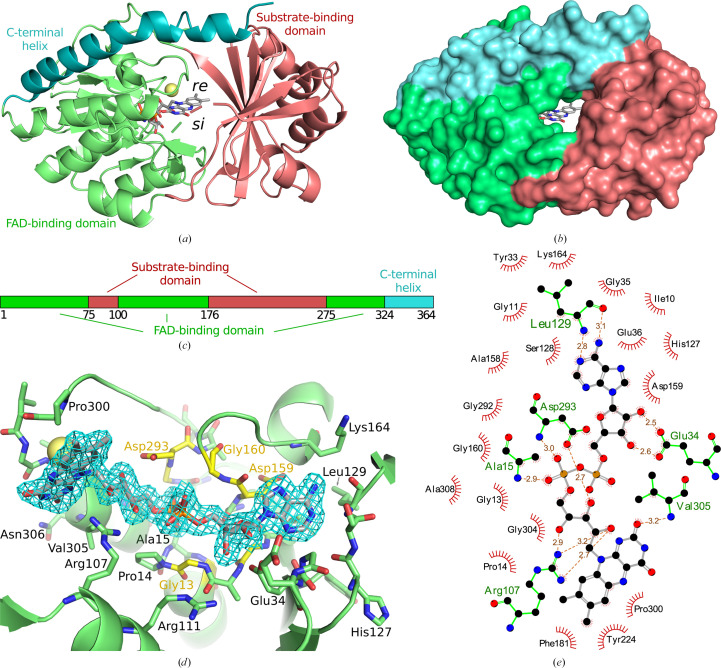
Structural analysis of *Sm*TetX. The substrate-binding domain is coloured red, the FAD-binding domain green and the C-terminal helix blue. C atoms of FAD are shown in grey in stick representation and a chloride anion is shown as a yellow sphere. (*a*, *b*) Overall fold represented in secondary structure (*a*) and surface (*b*) views along the access tunnel to the *re* site. (*c*) Schematic diagram of the *Sm*TetX domain structure; the numbers correspond to the native amino-acid sequence. (*d*) The binding of FAD in combined stick and secondary-structure representation: a view from the flavin *si* site. The 2*mF*_o_ − *DF*_c_ electron density is contoured in blue at the 1σ level. C atoms of the residues belonging to the G*X*G*XX*G, DG and GD binding motifs are coloured yellow. (*e*) Interactions of FAD with the protein. Hydrogen bonds are shown in orange with distances in Å. The graphics were prepared in *PyMOL* 2.5 (Schrödinger) and *LigPlot*+ (Laskowski & Swindells, 2011 ▸).

Crystals of *Sm*TetX provided severely anisotropic diffraction data. Nevertheless, the structure of the protein could be solved using molecular replacement and refined at 1.95 Å resolution. Anisotropy correction of the diffraction data (Tickle *et al.*, 2018 ▸) and the paired refinement protocol (Karplus & Diederichs, 2012 ▸; Malý *et al.*, 2020 ▸) proved to be beneficial for structure solution. The asymmetric unit consists of four *Sm*TetX molecules with pairwise r.m.s.d.s on C^α^ atoms below 0.34 Å; the substrate-binding site remains structurally unchanged across the individual protein chains. There are two pairs of identical covalent dimers cross-linked via two disulfide bridges, *i.e.* chains *A* and *B* are linked via Cys172(*A*)–Cys172(*B*) and Cys281(*A*)–Cys281(*B*) disulfide bridges. The enzyme shares its fold with the class A FAD-dependent monooxygenases (Paul *et al.*, 2021 ▸; Toplak *et al.*, 2021 ▸). The overall structure of the monomer is divided into a smaller substrate-binding domain (residues Glu75–Ser99 and Pro176–Tyr274) and a larger FAD-binding domain (Met1–His74, Lys100–Leu175 and Ser275–Pro323), which are stabilized by a long C-terminal α-helix (Asp324–Arg364) (Figs. 3 ▸ *a* and 3 ▸ *c*).

The substrate-binding domain is composed of a seven-stranded parallel β-sheet and four α-helices that are exposed to solvent. The FAD-binding domain consists of a three-stranded antiparallel β-sheet, a five-stranded parallel β-sheet, a two-stranded antiparallel β-sheet, six α-helices and one 3_10_-helix as analysed by *STRIDE* (Heinig & Frishman, 2004 ▸). Part of the Rossmann fold (βαβ motif) of the FAD-binding domain is located at the N-terminus and possesses the G*X*G*XX*G sequence motif (residues Gly11–Gly16) characteristic for binding the adenine moiety of FAD (Dym & Eisenberg, 2001 ▸; Kleiger & Eisenberg, 2002 ▸). The FAD adenine moiety is also bound by the previously described Asp159-Gly160 (DG) motif and the diphosphate moiety by the Gly292-Asp293 (GD) motif, similarly to other class A FDOs (Huijbers *et al.*, 2014 ▸; Paul *et al.*, 2021 ▸). The whole FAD molecule is well localized in electron density and is bound via 11 hydrogen bridges to Ala15, Glu34, Arg107, Leu129, Asp293 and Val305 (Figs. 3 ▸ *d* and 3 ▸ *e*).

The isoalloxazine moiety of FAD is accessible to solvent through two large cavities that are located between the substrate-binding and FAD-binding domains (Fig. 3 ▸ *b*). The binding sites in these cavities are further named according to their position with respect to the flavin ring as the *re* site (substrate-binding site) and the *si* site (NADPH-binding site). FAD is observed in the IN conformation in our structure, *i.e.* FAD is elongated and its isoalloxazine moiety reaches into the *re* site. A large portion of the FAD molecule is accessible to solvent and participates in the formation of the *si* site.

**Figure 4:**
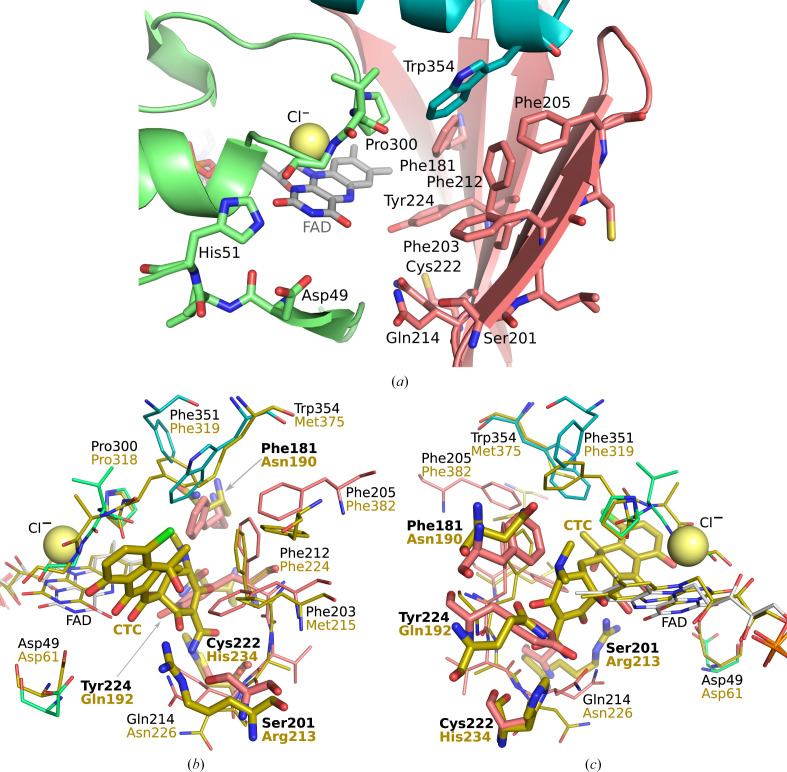
The active site of *Sm*TetX is located at the *re* site. (*a*) Composition of the active site in combined stick and secondary-structure representation. C atoms of the substrate-binding domain are coloured red, those of the FAD-binding domain green and those of the C-terminal helix blue. The chloride anion is shown as a yellow sphere. (*b*, *c*) Alignment of *Sm*TetX with the TetX–chlortetracycline (CTC) complex (Volkers *et al.*, 2011 ▸; PDB entry 2y6r) in yellow shown in stick representation. The r.m.s.d. was 3.74 Å using 284 C^α^ atoms of chains *A*. The graphics were prepared in *PyMOL* 2.5 (Schrödinger).

The *re* site consists of the main chain of the loop Pro300–Gly302 and the side chains of a large nonpolar aromatic region (Phe181, Phe203, Phe205, Phe212, Tyr224 and Trp354), a polar region (Ser201, Gln214 and Cys222) and Asp49 (Fig. 4 ▸ *a*). A chloride anion was modelled in the *re* site near the FAD isoalloxazine moiety according to the signal strength in the 2*mF*_o_ − *DF*_c_ map, the typical distances to adjacent atoms and their chemical properties. It is bound at the pyrimidine moiety of isoalloxazine and coordinated by the main-chain N atoms of the Pro300–Val303 loop. Several residues at the protein termini (Met1-Gln2, Gly358–Gly364) and the *re* site (Gly93–Asp101) could not be modelled due to a lack of electron density.

### 3.4. Docking of antibiotic representatives

In order to inspect the interactions of the protein with likely ligands, *in silico* ligand docking of selected tetracycline molecules (Supplementary Fig. S6) and other antibiotic representatives into the binding *re* site were carried out (Table 2 ▸). As these ligands can appear in multiple tautomeric forms, we took the keto (C3)–amino (C4) tautomerism into account. However, we also tested the enolate (C3)–ammonium (C4) tautomerism of tetracycline (Aleksandrov *et al.*, 2007 ▸) and obtained similar results both in terms of relative energies in the unbound solvated state at the RI-TPSS-D3/TZVP/CPCM level of theory as well as in the predicted binding affinity to the studied protein. The molecule of anhydrotetracycline was docked to act as a potential substrate (*Sm*TetX in FAD-IN conformation), similarly to as observed in the complex with TetX6 (Kumar *et al.*, 2023 ▸); nevertheless, it should be noted that anhydrotetracycline inhibits Tet(50) when bound in the *re* site with the FAD-OUT conformation (Park *et al.*, 2017 ▸). The chloride anion present in the *re* site in our crystal structure was taken into account in the calculations.

## 4. Discussion

*Sm*TetX shows activity against oxytetracycline (OTC; Fig. 2 ▸), which would classify the enzyme as a member of the tetracycline destructase family (Cheng *et al.*, 2022 ▸; Markley & Wencewicz, 2018 ▸). Our estimated turnover number of *Sm*TetX towards OTC as a substrate (2.6 ± 0.7 s^−1^) is comparable to that of the tetracycline destructase TetX2 (1.3 ± 0.1 s^−1^; Yang *et al.*, 2004 ▸) and that of TetX4 towards tigecycline (2.03 ± 0.03 s^−1^; Cheng *et al.*, 2021 ▸). However, all of the described tetracycline destructases strictly require NADPH for their catalytic activity. *Sm*TetX oxidizes the NADPH co-factor in the absence of OTC (Figs. 2 ▸ *c* and 2 ▸ *i*). This is consistent within the family as NADPH reduces FAD prior to the formation of C4a-hydroperoxyflavin, which is needed for electrophilic attack on a tetracycline substrate. Remarkably, the changes in the OTC absorption spectra for *Sm*TetX occurred even in the absence of NADPH (Figs. 2 ▸ *a* and 2 ▸ *g*). The reactions reach saturation before substrate exhaustion. Both observations can be explained by the partial presence of the reduced state of FAD in *Sm*TetX as indicated by the UV–Vis absorption spectrum (Supplementary Fig. S3).

The overall fold of *Sm*TetX is very similar to those of the tetracycline destructases TetX (Volkers *et al.*, 2011 ▸) and TetX2 (Walkiewicz *et al.*, 2011 ▸) from *B. thetaiotaomicron*, TetX4 from *E. coli* (Cheng *et al.*, 2021 ▸) and TetX7 from *P. aeruginosa* (Gasparrini *et al.*, 2020 ▸).

The crystal structure contains *Sm*TetX in the form of covalent dimers, which are most likely to be artefacts of crystallization. Other experimental methods (SEC and SAXS; Supplementary Fig. S5) indicate a monomeric state of the protein in solution, and mass spectrometry confirms the absence of disulfide bridges. The *Sm*TetX monomer organization observed in our structure is very close to that of similar enzymes. Thus, the covalent links between the *Sm*TetX chains very likely do not affect the structure of the *Sm*TetX monomer itself. We assume that *Sm*TetX acts as a monomer in solution and not as a dimer as found in our crystal structure.

The binding of FAD to *Sm*TetX is conserved within the family of class A FMOs. In our case, we only observe the IN conformation of FAD; however, the size of the *si* site would also allow the accommodation of the bent OUT conformation that was observed in the crystal structure of Tet(50) (Park *et al.*, 2017 ▸).

Despite numerous co-crystallization and soaking trials, our attempts to experimentally determine complexes of *Sm*TetX with tetracycline antibiotics or co-factors failed. Therefore, we identified putative binding sites according to the structures of similar complexes. We hypothesize that the *si* site is the binding site for NADPH as in the case of 4-hydroxybenzoate hydroxylase, which is the most thoroughly studied class A FMO (Crozier-Reabe & Moran, 2012 ▸; Eppink *et al.*, 1998 ▸, 1999 ▸). On the basis of structures of tetracycline destructases in complex with tetracyclines (Cheng *et al.*, 2022 ▸; Park *et al.*, 2017 ▸; Volkers *et al.*, 2011 ▸, 2013 ▸), we hypothesize that the substrate-binding site is located in the *re* site (Fig. 4 ▸ *a*).

**Figure 5:**
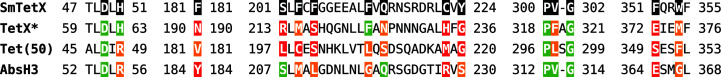
Multiple structure-based sequence alignment of *Sm*TetX with the tetracycline destructases TetX from *B. thetaiotaomicron* (PDB entry 2y6r; Volkers *et al.*, 2011 ▸) and Tet(50) (PDB entry 5tue; Park *et al.*, 2017 ▸), and the putative reductase AbsH3 from *Streptomyces* sp. (PDB entry 6n04; Clinger *et al.*, 2021 ▸). Regions significant for the *re* site of the enzyme are shown. The key residues in the binding pocket of *Sm*TetX are highlighted by a black background (and shown in Fig. 4 ▸). Identity, similarity and dissimilarity of the key amino acids are marked with green, orange and red backgrounds, respectively. *The sequence and structure of this region are the same for TetX, TetX2, TetX4 and TetX7 (Cheng *et al.*, 2022 ▸).

The destructases TetX and Tet(50) and the putative reductase AbsH3 from the abyssomicin biosynthesis pathway (used in molecular replacement; Clinger *et al.*, 2021 ▸; PDB entry 6n04) are the most similar enzymes in terms of sequence, overall structure and structure of the active site. Structure-based sequence alignment (Fig. 5 ▸) reveals many similarities in the substrate-binding site as well as some unique features of *Sm*TetX. The most conserved parts are regions Thr47–Leu50 (*Sm*TetX numbering), which is placed next to the pyrimidine moiety of isoalloxazine, and Pro300–Gly302, which is located above the *re* side of the isoalloxazine ring.

Further discussion is based on a structural comparison of *Sm*TetX with TetX, the most similar tetracycline destructase with a thoroughly described structure and function. Several important residues are identical in *Sm*TetX and TetX, *i.e.* Asp49, His51, Phe212, Pro300 and Gly302 (*Sm*TetX numbering). The side chains of Phe205 and Phe351 in *Sm*TetX correspond to their phenylalanine analogues in TetX, but they originate from different secondary-structure elements (Figs. 4 ▸ *b* and 5 ▸ *c*). The aromatic residues Phe203 and Trp354 in *Sm*TetX replace Met203 and Met375 in TetX to conserve the nonpolar character of these sites. The major difference in *Sm*TetX are residues Phe181 and Tyr224, which expose their aromatic side chains to the *re* site and thus change the nature of the active site, in contrast to Asn190 and Gln192 in TetX, which participate in substrate binding (PDB entry 2y6r). Moreover, Cys222 and Ser201 in *Sm*TetX replace His234 and Arg213, respectively, in TetX. Overall, of the 15 residues forming the substrate-binding pocket of *Sm*TetX, five are identical, four have a similar character and six have a significantly different character. The differences raise questions about the actual binding of tetracycline antibiotics, *i.e.* whether the *re* site allows the accommodation of such ligands and whether their binding modes are compatible with the known tetracycline modification sites reported for tetracycline destructases.

Due to the lack of a structure of *Sm*TetX in complex with a ligand, we attempted to investigate the binding of tetracycline representatives (tetracycline, oxytetracycline, anhydrotetra­cycline and chlortetracycline) by the use of computational ligand docking. The calculations could be influenced by the unmodelled region at the entrance to the *re* site (Gly93–Asp101) in the *Sm*TetX crystal structure (Fig. 3 ▸ *a*). However, the amino-acid sequence and length of this region are similar to those of TetX, where this loop is not directly involved in ligand binding, for example in the TetX–chlortetracycline complex (PDB entry 2y6r). Therefore, we assume that the missing residues do not affect the docking results for molecules of the size of tetracycline antibiotics and smaller.

In the docking study, tetracycline representatives were among the highest affinity binders, for example anhydro­tetracycline (–9.1 kcal mol^−1^) and OTC (–8.7 kcal mol^−1^) (Table 2 ▸ and Supplementary Fig. S6). The side chains of Phe181 and Tyr224 of *Sm*TetX, representing the largest difference in the active site with respect to TetX, modify the bottom of the binding cavity and lead to a small shift of the docked ligands compared, for example, with the TetX–chlortetracycline complex (Figs. 4 ▸ *b* and 4 ▸ *c*). Nevertheless, the calculated poses of OTC place its C atoms that are susceptible to modification by tetracycline destructases, *i.e.* C1, C2, C3 and C11a, in the vicinity of the reactive C4a of FAD (Supplementary Figs. S6 and S7). Recently, a structure of Tet(X6) in complex with anhydrotetracycline bound in a substrate-like orientation was published (PDB entry 8er0; Kumar *et al.*, 2023 ▸). In comparison with our docking of this ligand, the positions of the A and D rings (Fig. 1 ▸) are swapped.

Apart from tetracycline antibiotics, ampicillin, rifampin and streptomycin were also shown to be high-affinity binders. Interestingly, the class A FAD-dependent monooxygenases include the rifamycin-inactivating enzyme Rox from *Streptomyces venezuelae* (PDB entries 5vqb and 6brd; Koteva *et al.*, 2018 ▸) that is capable of rifampin inactivation. In comparison with the tetracycline destructases, the Rox structure contains a larger active site and two additional consecutive α-helices.

Gln214 of *Sm*TetX lining the outer edge of the *re* site is found in alternative conformations in the structure, suggesting that it possesses flexibility that might be involved in ligand binding. Interestingly, in the flexible side-chain docking calculations, a twist of the glutamine side chain (Supplementary Fig. S7) was indicated to a conformation analogous to Asn226 in the TetX–chlortetracycline complex (PDB entry 2y6r; Figs. 4 ▸ *b* and 4 ▸ *c*). These docking results show two possible binding modes of OTC: both are compatible with enzymatic modification and they differ in the location of the A and D rings of OTC.

Therefore, the *re* site of *Sm*TetX is capable of accommodating tetracycline antibiotics and the suggested poses would enable electrophilic attack of hydroperoxyflavin on the tetracycline ring.

## 5. Conclusion

Several clinical isolates of *S. maltophilia* contain a reported FAD-dependent monooxygenase similar to the tetracycline destructase TetX from *B. thetaiotaomicron*. We have shown that this enzyme is capable of activity towards oxytetracycline and that its structure is very similar to that of TetX. *Sm*TetX has a unique structural feature in the active site within this enzyme family: a large aromatic region formed by Phe181, Phe203, Phe205, Phe212, Tyr224 and Trp354. According to our *in silico* binding analysis, despite the remarkable differences in the active site, tetracycline representatives are among the highest affinity binders of the tested antibiotics and the aromatic region probably participates in tetracycline binding. Furthermore, the rate of oxytetracycline degradation is comparable to the rates shown by other destructases. Hence, these results provide evidence that *S. maltophilia* codes for an enzyme that is capable of degrading tetracycline antibiotics, even if such a mechanism is not essential for its resistance to these antibiotics.

## 6. Related literature

The following references are cited in the supporting information for this article: Islam *et al.* (2013 ▸) and Schwinn *et al.* (2020 ▸).

## Supplementary Material

PDB reference: *Sm*TetX, 8aq8

SASBDB reference: *Sm*TetX, without reducing agent, SASDPV7

SASBDB reference: with reducing agent, SASDPV7

Supplementary Figures and Table. DOI: 10.1107/S2053230X23005381/va5051sup1.pdf

Raw diffraction images for PDB entry 8aq8.: https://doi.org/10.15785/SBGRID/956

Raw scattering images for SASBDB entries SASDPV7 and SASDPW7.: https://doi.org/10.5281/zenodo.7348780

## Figures and Tables

**Table 1:** Data-collection and merging statistics (after anisotropy correction) and structure-refinement parameters for *Sm*TetX Values in parentheses are for the highest resolution shell.

PDB entry	8aq8
Data collection	
X-ray source	BL14.1, BESSY II
Wavelength (Å)	0.9180
Detector	PILATUS 6M
Temperature (K)	100
Crystal-to-detector distance (mm)	424
No. of oscillation images	2400
Exposure time per image (s)	0.1
Oscillation width (°)	0.1
Data processing	
Space group	*P*2_1_
*a*, *b*, *c* (Å)	52.9, 160.5, 95.6
α, β, γ (°)	90, 95.9, 90
Resolution (Å)	48.21–1.95 (2.01–1.95)
No. of reflections	467117 (25410)
No. of unique reflections	98291 (4915)
Multiplicity	4.8 (5.2)
Completeness (spherical) (%)	85.3 (48.5)
Completeness (ellipsoidal) (%)	96.3 (92.9)
*R*_meas_	0.156 (1.702)
*R*_p.i.m._	0.070 (0.729)
Mean *I*/σ(*I*)	8.6 (1.1)
CC_1/2_	0.997 (0.378)
CC*	0.999 (0.741)
Mosaicity (°)	0.2
Solvent content (%)	52.6
Refinement	
*R*_work_	0.2002 (0.3185)
*R*_free_ (5% reflections)	0.2410 (0.3289)
*R*_all_	0.2022 (0.3175)
CC_work_	0.964 (0.592)
CC_free_	0.938 (0.617)
Mean ADP (Å^2^)	28.9
No. of protein chains in asymmetric unit	4
No. of atoms	11859
No. of water molecules	1111
Ligands	4 × FAD, 6 × ethylene glycol, 2 × polyethylene glycol, 3 × 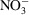 , 2 × 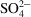 , 4 × Cl^−^
R.m.s.d. from ideal bond lengths (Å)	0.009
R.m.s.d. from ideal angles (°)	1.498
Ramachandran favoured (%)	97
Ramachandran outliers (%)	0
Rama-*Z* score (Sobolev *et al.*, 2020 ▸)	−1.3 ± 0.2

**Table 2:** The lowest calculated binding energies of selected antibiotics in the binding *re* site determined by molecular docking Tetracycline representatives are labelled with an asterisk.

Antibiotic molecule	Affinity (kcal mol^−1^)
*Anhydrotetracycline	−9.08
Ampicillin	−8.81
*Oxytetracycline	−8.70
*Tetracycline	−8.65
Rifampin	−8.58
Streptomycin	−8.41
*Chlortetracycline	−8.17
Levocetirizine	−8.03
Amoxicillin	−7.83
Amikacin	−7.79
Erythromycin	−7.69
Mitomycin	−7.67
Chloramphenicol	−7.62
Paromomycin	−7.48
Rifamycin SV	−7.39
Lincomycin	−7.38
Abyssomicin C	−7.38
Kanamycin	−7.38
Clarithromycin	−6.95
Gentamicin	−6.83
Metronidazole	−5.31
