# Supplementary material for: Tetracycline-modifying enzyme SmTetX from Stenotrophomonas maltophilia
Source: Acta Crystallogr F Struct Biol Commun. 2023 Jul 5;79(Pt 7):180–92. doi: 10.1107/S2053230X23005381 (PMC10327574; doi:10.1107/S2053230X23005381)
Supplement: Supplementary file 1 [file f-79-00180-sup1.pdf]

Acta Cryst F
STRUCTURAL BIOLOGY
COMMUNICATIONS

Volume 79 (2023)

Supporting information for article:

Tetracycline-modifying enzyme *Sm*TetX from *Stenotrophomonas maltophilia*

Martin Malý, Petr Kolenko, Jan Stránský, Leona Švecová, Jarmila Dušková, Tomáš Kovaľ, Tereza Skálová, Mária Trundová, Kristýna Adámková, Jiří Černý, Paulína Božíková and Jan Dohnálek

construct used for experiments

```
                              1                                        39
MGSSHHHHHH SSGENLYFQG HMQHPPRIAV IGAGPAGLTA ALILHRGGHR VSVYEGEASG
    6xHis-tag
         TEV-cleavage site   the first residue observed in the crystal structure

40                                                                     99
QQRTQGGTLD LHDDSGQVAL QRAGLLEAFR AVARHEGQEA RMGDPWSGEI TTGGFGPEGS
                                                 not observed in the crystal structure

100                                                                   159
KDKPEIDRGD LRQLLLDALP ADTVQWGHSL TALVSAQANG HGLRFANGVQ READIVIGAD

160                                                                   219
GAWSKVRAAL STCQPLPTGI TFFEGWIAQP AADIAAMVGQ GSLFCFGGEE ALFVQRNSRD

220                                                                   279
RLCVYAALKR SSEWLDAQIA QHGIRTLVTG CYTGWATNLR RLLEACTDFV RRPIYSLPAD

280                                                                   339
FCWTPRDGVT LIGDAAHLMP PVGVGVNLAM LDASDVAMAL CARPDAISAM RDAEVLVRER

340                     364
ARALMPDAIE GFQRWFITGG DAPRG
   the last residue observed
      in the crystal structure
```

Figure S1 Amino acid sequence of the used construct of *Sm*TetX.

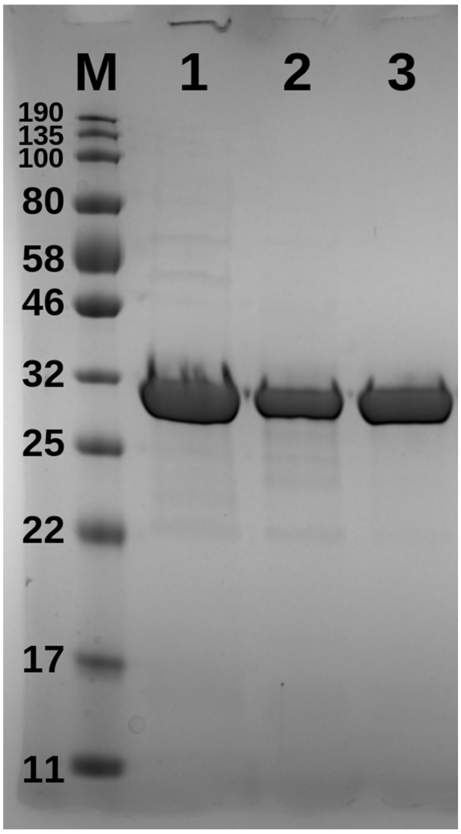

Figure S2 Sodium dodecyl sulfate-polyacrylamide gel electrophoresis of *Sm*TetX. Lane content: marker (M); *Sm*TetX after tag cleavage (1); *Sm*TetX after tag cleavage, SEC fraction 1 (2); *Sm*TetX after tag cleavage, SEC fraction 2, which was used for the further experiments (3). The gel was photographed with an Azure 300 (Azure Biosystems).

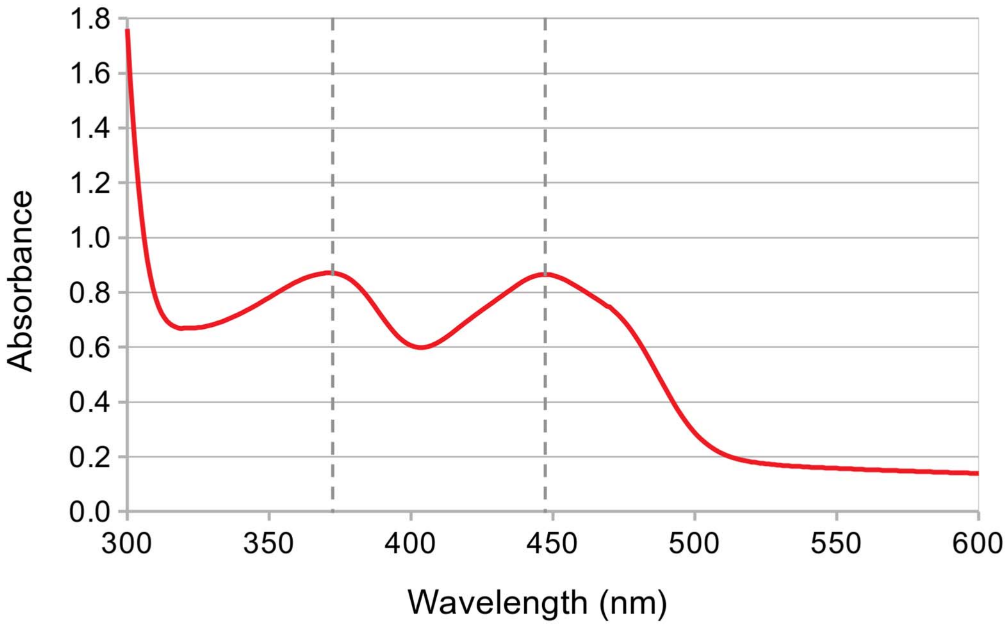

Figure S3 UV-VIS spectrum of *Sm*TetX in a concentration of 3 mg ml^{-1} measured in 100 mM TAPS, pH 8.5 in a quartz cuvette (optical path 10 mm). Maxima corresponding to FAD absorption, shown as dashed lines at 370 nm and 447 nm, possess almost equal height. Nevertheless, FAD itself in the oxidized state has a significantly higher maximum at 447 nm than at 370 nm; whereas FAD itself in the reduced state absorbs very weakly at 447 nm (Crozier-Reabe & Moran, 2012; Islam *et al.*, 2013; Schwinn *et al.*, 2020). Thus, FAD in *Sm*TetX is dominantly in the oxidized state but the reduced state is likely partially present as well.

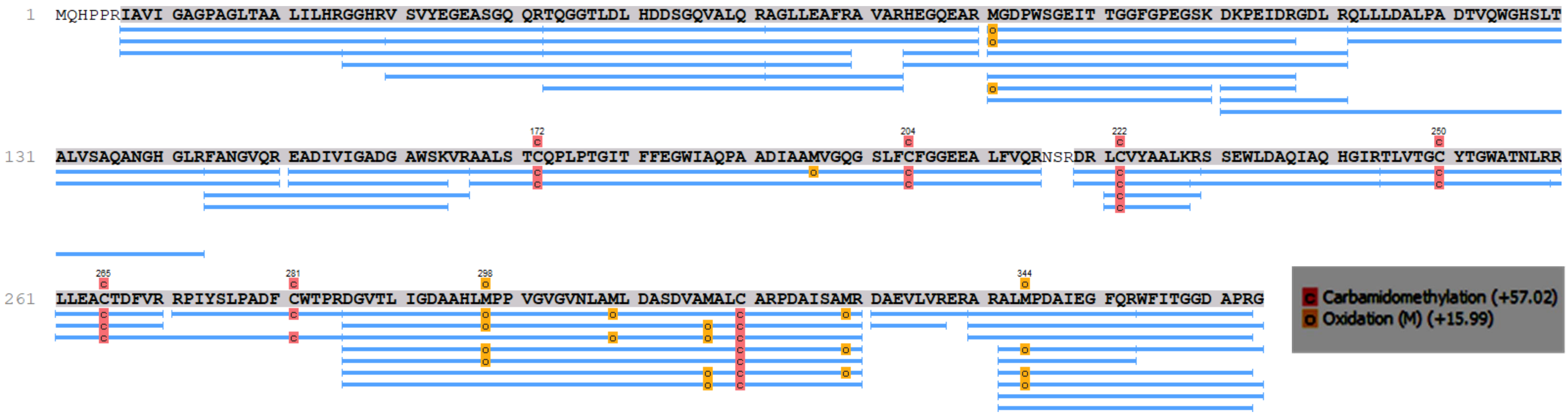

Figure S4 Mass spectrometry analysis of *Sm*TetX after fragmentation to peptides by trypsin. Blue lines represent individual detected fragments. Carbamidomethylated cysteine residues (additional mass 57.02 Da) are labelled in red and oxidized methionine residues (additional mass 15.99 Da) in orange. The *Sm*TetX sequence is basically completely covered by the individual peptide fragments. No disulphide bridges are present as all the cysteine residues were observed alkylated.

Table S1 Small angle X-ray scattering of *Sm*TetX: parameters of data collection and processing.

Sample details	with 30 mM DTT	without DTT
SASBDB accession code	SASDPW7	SASDPV7
Buffer	25 mM Bis-Tris, 150 mM NaCl, pH 6.5, 30 mM DTT	25 mM Bis-Tris, 150 mM NaCl, pH 6.5
Theoretical molecular weight (Da)	38941	
Concentration of the sample (mg ml^{-1})	2.4	
SAXS data-collection parameters		
Instrument	Excillum MetalJet C2+ X-ray source with Anton Paar SAXSpoint 2.0 and Dectris EIGER R 1M detector	
X-ray wavelength (Å)	1.34	
Beam size at sample (mm)	0.94	
Beamstop size (mm)	2	
Sample to detector distance (mm)	826	
Measurement q range (nm^{-1})	0.07–4.14	
Sample temperature (°C)	4	
Exposure time (s)	90x16	61x16
Parameters of conversion to absolutes scale	Calibrated using water as scattering standard	
Guinier analysis (*AUTORG*)		
R_g (nm)	2.36 ± 0.03	2.36 ± 0.04
$I(0)$ (cm^{-2})	0.153 ± 0.001	0.135 ± 0.001
Point index, of the Guinier region	9–82	18–82
q range (nm^{-1})	0.14–0.55	0.18–0.55
Fidelity	0.97	0.75
Aggregation index	0.03	0.25
Analysis using indirect fourier transform (*GNOM*)		
R_g (nm)	2.28 ± 0.01	2.32 ± 0.02
$I(0)$ (cm^{-2})	0.1501 ± 0.0009	0.133 ± 0.001
q range (nm^{-1})	0.13–1.80	0.18–1.80
Real space range D_{min}–D_{max} (nm)	0.0–6.4	0.0–6.8
Fit quality	0.79	0.74
Ambiguity score	1.15	0.95
Shannon analysis (*SHANUM*)		
Optimal Shannon channels	7	8
Optimal q_{max} (nm^{-1})	3.10	3.44
Molecular weight estimates		
M_W from Porod Volume		
V_P (nm^3)	22.57	18.19
M_W (Da)	14107	11371
M_W from Porod Invariant		
M_W (Da)	27961	35544
M_W from Volume of Correlation		
V_C (nm^3)	309.86	319.07
M_W (Da)	33057	35057
M_W from Apparent Volume		

Q (nm^{-3})	0.275	0.257
V_{app} (nm^3)	71.77	76.76
M_W (Da)	35731	38559
M_W from Size and Shape		
M_W (Da)	37793	40146
M_W from Bayesian inference		
M_W (Da)	33100	36900
M_W score	0.28	0.47
Agreement between the data and the atomic model (*CRYSOL*)		
M_W from structure on input (Da)	37980	37980
q range (nm^{-1})	0.1–1.5	0.1–1.5
Predicted R_g (nm)	2.25	2.27
χ^2	0.90	1.05
Results of *ab-initio* shape determination		
Particle symmetry	*P*1	*P*1
Particle anisometry	Unknown	Unknown
q range (nm^{-1})	0.13–1.80	0.18–1.80
DAMMIF		
M_W (Da)	35000 ± 100	36700 ± 100
Particle R_g (nm)	2.2805 ± 0.0002	2.3201 ± 0.0002
Particle D_{max} (nm)	8.0 ± 0.1	9.1 ± 0.5
χ^2	0.8129 ± 0.0007	0.8541 ± 0.0003
Number of models on input	32	32
No. of excluded models	2	2
DAMMIN		
Particle R_g (nm)	2.29	2.33
Particle D_{max} (nm)	7.33	7.76
χ^2	0.80	0.84

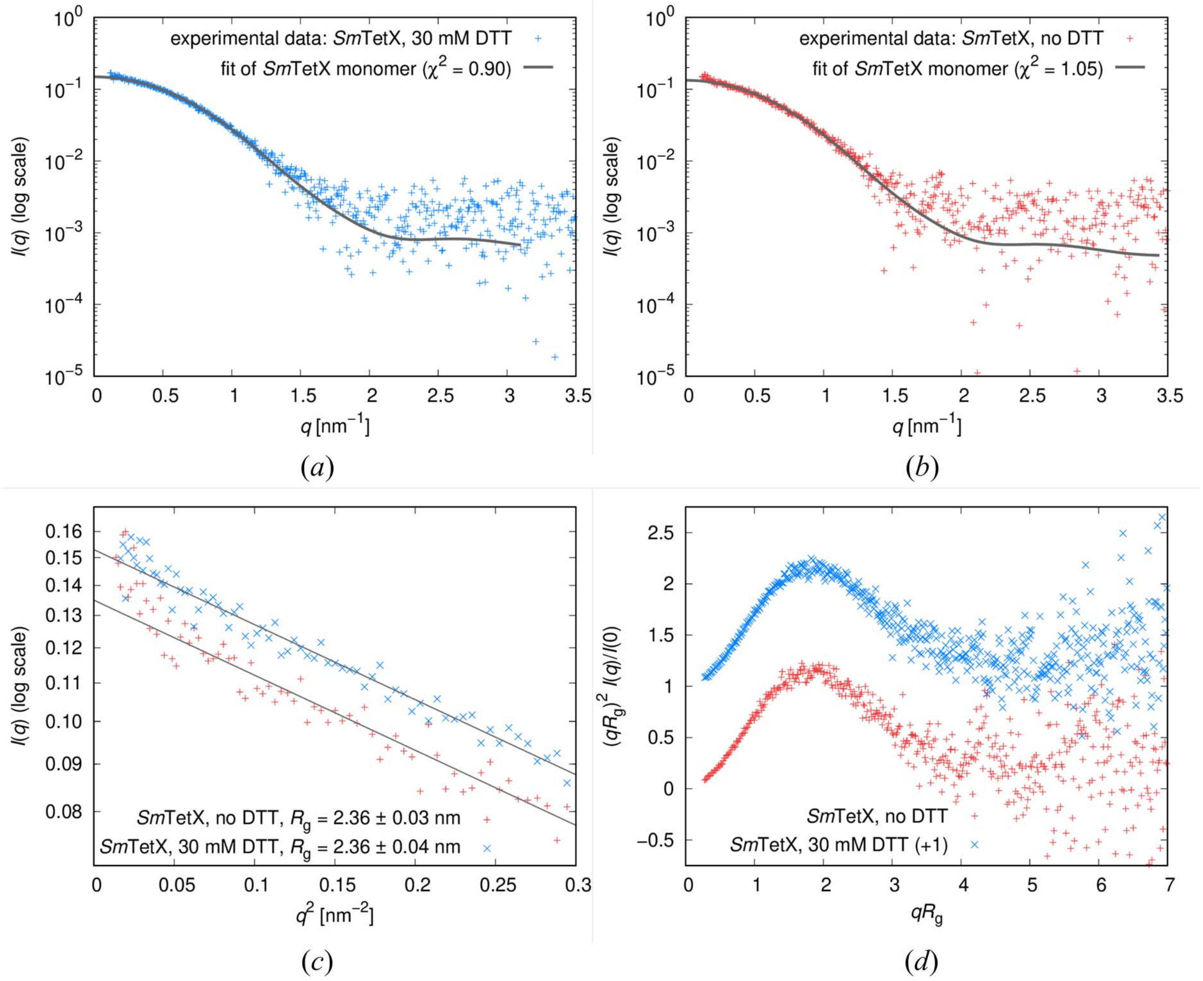

Figure S5 SAXS analysis of *Sm*TetX: Intensities after reduction for the sample with 30 mM dithiotreitol (DTT) (*a*, blue) and without the reducing agent (*b*, red); theoretical scattering plots computed with *CRYSOL* for the monomer from the crystal structure, plotted in grey, fit well the measured data (χ^2 0.90 and 1.05), whereas those for the covalently linked dimer result in unacceptably high $\chi^2 > 5$. (*c*) Guinier plots with fits. (*d*) Dimensionless Kratky plots possess the shape typical for globular proteins, the data for the sample with DTT are plotted with a shift (+1 in the *y* axis).

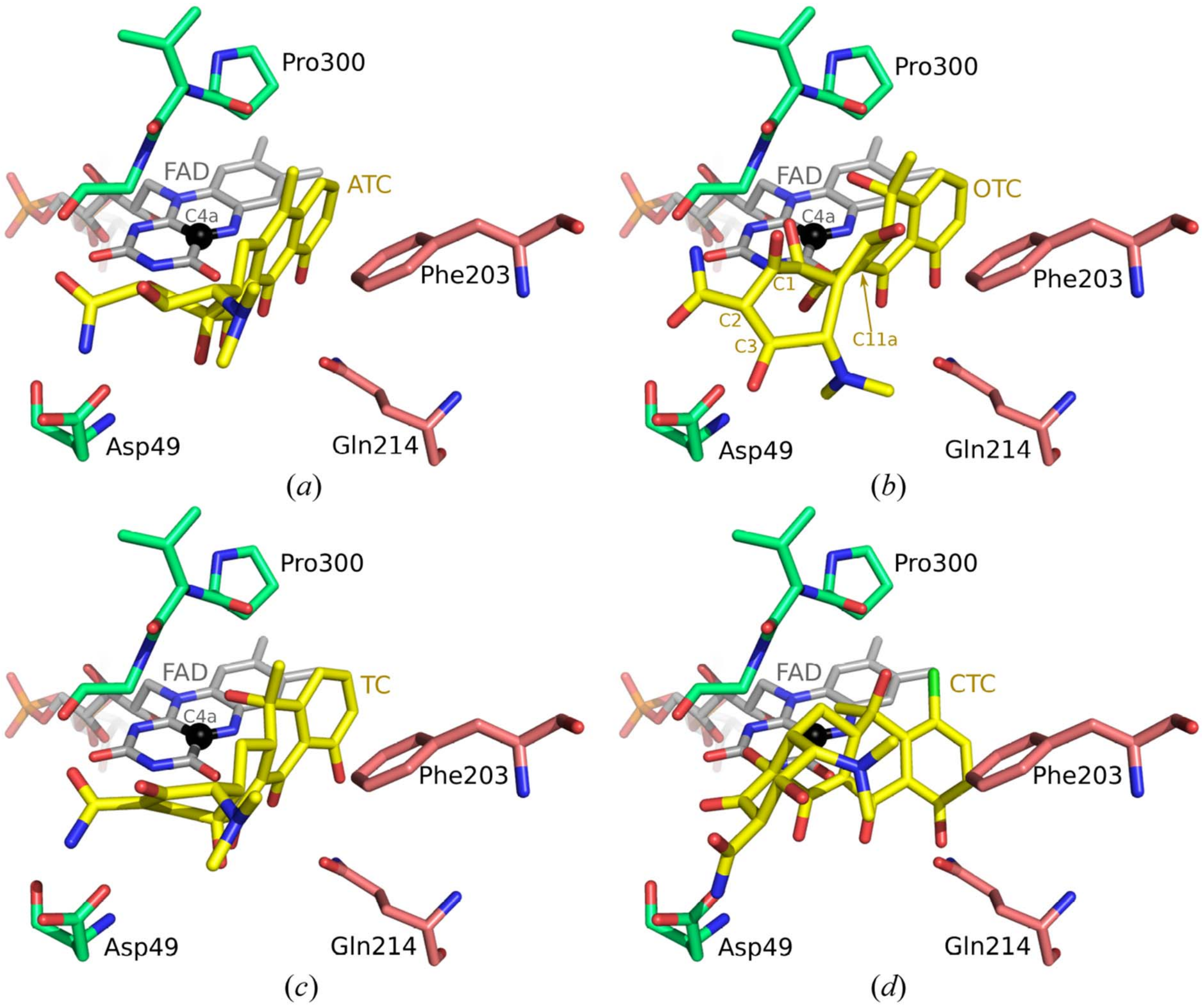

Figure S6 Docking of tetracycline representatives in the *re*-site. Positions of side chains were rigid during calculation. Corresponding affinities as calculated in *Autodock Vina* (Eberhardt *et al.*, 2021; Trott & Olson, 2009) are listed in brackets. (*a*) anhydrotetracycline (ATC; –9.01 kcal mol^{-1}), (*b*) oxy-tetracycline (OTC; –8.70 kcal mol^{-1}), (*c*) tetracycline (TC; –8.65 kcal mol^{-1}), (*d*) chlortetracycline (CTC; –8.17 kcal mol^{-1}). The C4a atom of FAD that is expected to be modified by the hydroperoxy group is highlighted as a black sphere and the atoms of OTC most likely modified by tetracycline de-structases are labelled. The graphics were prepared in *PyMOL* 2.5 (Schrödinger).

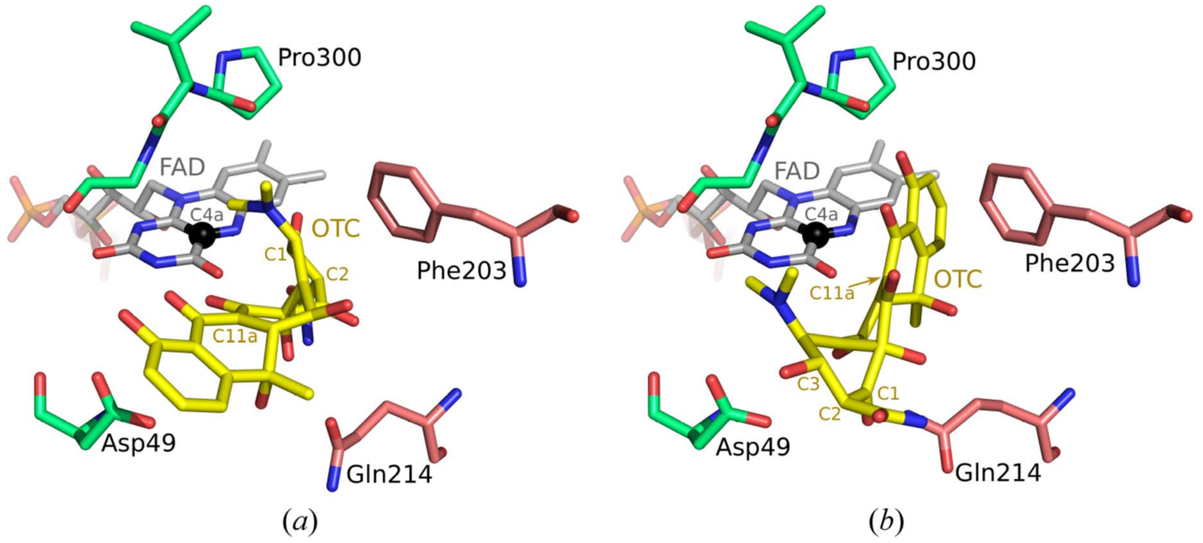

Figure S7 Docking of oxytetracycline in the *re*-site using flexible side chains. The corresponding affinities as calculated in *Autodock Vina* (Eberhardt *et al.*, 2021; Trott & Olson, 2009) are: (*a*) –11.6 kcal mol^{-1}, (*b*) –11.5 kcal mol^{-1}. The C4a atom of FAD that is expected to be modified by the hydroperoxy group is highlighted as a black sphere and the atoms of OTC most likely modified by tetracycline destructases are labelled. The graphics were prepared in *PyMOL* 2.5 (Schrödinger).
